# Granzyme B PET Imaging Uncovers Dynamic Patterns of Disease Activity and Therapeutic Response in a Murine Colitis Model

**DOI:** 10.3390/ijms27104194

**Published:** 2026-05-08

**Authors:** Arvin Haj-Mirzaian, Madeline Ma, Nicole Hofmann, Hushan Yuan, Umar Mahmood, Pedram Heidari

**Affiliations:** Center for Precision Imaging, Division of Nuclear Medicine and Molecular Imaging, Department of Radiology, Massachusetts General Hospital, Harvard Medical School, Boston, MA 02129, USAnhofmann19@icloud.com (N.H.);

**Keywords:** granzyme B, colitis, inflammatory bowel disease, PET imaging, ^68^Ga-NOTA-GZP, anti-TNF, prednisolone, anti-IL-23

## Abstract

The evaluation of therapeutic response is essential in disease monitoring both for disease status and treatment efficacy in inflammatory bowel disease. Here, we focused on the use of positron emission tomography directed towards granzyme B, a serine protease released by activated cytotoxic T cells and natural killer cells, to evaluate the dynamics of therapeutic response in a colitis model. The goal was to explore the use of granzyme B positron emission tomography as a non-invasive biomarker to monitor disease activity and therapeutic response across several treatments in a dextran sulfate sodium-induced colitis model. C57BL/6 interleukin-10 knockout mice were divided into five groups, including a negative control, positive control and three treatment arms (antitumor necrosis factor, prednisolone, and anti-interleukin-23). The negative control group received regular water, while all other groups were induced with colitis via 3% DSS water for 1 week followed by normal water. Treatments were initiated after colitis was induced (anti-TNF antibody, prednisolone, or anti-IL-23 antibody). Positron emission tomography/computed tomography imaging with 68Ga-NOTA-GZP was performed at baseline (after colitis induction, before therapy), and at 1 and 2 weeks after treatment initiation. Histological analyses were also performed at 1 and 2 weeks after treatment initiation. *Gzmb* expression and histological changes were also assessed with immunofluorescence staining and bulk ribonucleic acid sequencing. *Gzmb*-targeted PET imaging revealed distinct longitudinal patterns of colonic tracer uptake related to treatment response. In positive control mice with DSS colitis (no treatment), bowel uptake of ^68^Ga-NOTA-GZP increased significantly from baseline to week 2. Anti-TNF treatment reduced granzyme B positron emission tomography uptake significantly at week 2, approaching levels seen in negative controls. In prednisolone-treated mice, ^68^Ga-NOTA-GZP uptake decreased at week 1 but rose significantly by week 2 but still was in normal range. Anti-IL-23 therapy produced a significantly elevated *Gzmb* PET signal at week 1, followed by a significant decline by week 2 of treatment. The imaging trends were corroborated by tissue analyses and IF staining for *Gzmb*, which revealed no colonic expression in negative controls and strong *Gzmb* elevation in positive controls and the prednisolone group but a decreased *Gzmb* signal in the anti-TNF and late anti-IL-23 groups. Bulk RNA sequencing also supported these findings, with *Gzmb* gene expression tracking with inflammation severity and NK/T cell abundance and decreasing after effective therapy. *Gzmb*-targeted PET/CT allows for dynamic and non-invasive assessment of intestinal immune compartment activity and an assessment of therapy in colitis. *Gzmb* PET was able to detect initial treatment responses of anti-TNF, steroid and anti-IL-23 based on changes in the *Gzmb* PET signal. This suggests that clinical *Gzmb* PET imaging may serve as precision imaging for monitoring disease activity with treatment in IBD and help improve patient care by identifying responders and non-responders in real time.

## 1. Introduction

Inflammatory bowel disease (IBD), including Crohn’s disease (CD) and ulcerative colitis (UC), is a chronic, is a relapsing inflammatory disease of the gut affecting millions across the globe [1]. Despite relatively rapid advances in therapy over the last twenty years, IBD still poses major clinical challenges, particularly when it comes to objectively quantifying disease activity and evaluating treatment response in a timely manner [2,3]. The heterogeneous phenomena observed in patients with IBD, together with patient-specific therapy responses and drug effects, point to a need for precision approaches [4].

Current methods of monitoring IBD activity have significant shortcomings; colonoscopy remains the gold standard for detection of mucosal inflammation and mucosal healing and is routinely used for longitudinal disease surveillance on an annual or biannual basis. However, colonoscopy is invasive, carries procedural risks, and provides primarily structural and visual assessment of the mucosa, which may not fully capture underlying immune activity. Moreover, colonoscopy may not be practical for more frequent assessments, such as early evaluation of treatment response within weeks of therapy initiation, or for evaluating disease segments beyond its reach in patients with proximal small bowel Crohn’s disease [3,5,6]. Serum and fecal biomarkers such as C-reactive protein (CRP) and fecal calprotectin offer an alternative means of non-invasive detection but are not specific and cannot localize the disease [7]. The situation is exacerbated with the multitude of therapies available for IBD, including biologics targeting TNF, IL-23, integrins and small-molecule inhibitors [8]. The ability to quickly differentiate patients that are unlikely to respond from those that will optimizes treatment, decreasing the possibility of disease progression and complications [9].

PET/CT could offer non-invasive means of monitoring inflammatory processes. While routine anatomical imaging with MRI or CT can identify structural changes, they frequently miss early evidence of immunologic activity [10,11,12]. While ^18^F-fluorodeoxyglucose (FDG) PET has been shown to be helpful for detecting active IBD, its clinical utility is limited by nonspecific physiologic uptake in metabolically active tissues, as FDG cannot distinguish immune-related inflammation from other metabolically active processes such as infection, malignancy, or physiologic bowel activity [11]. These considerations have spurred interest in engineering novel PET tracers targeting specific immune biomarkers that drive IBD pathology, with the potential to provide complementary immunological information beyond what metabolic imaging alone can offer. Granzyme B (Gzmb) represents a particularly appealing focus for molecular imaging of intestinal inflammation. A serine protease stored in the cytotoxic granules of CD8^+^ T-lymphocytes and natural killer (NK) cells, Gzmb is released following immune activation and elicits target cell apoptosis [13]. In addition to its classical cytotoxic function, Gzmb also exerts pro-inflammatory effects by cleaving extracellular matrix components and activating cytokines that assist in perpetuating an inflammatory cascade [13]. Of note, Gzmb expression levels are markedly increased during active IBD, with clinical studies showing significantly higher levels of mucosal Gzmb in patients with active UC and CD compared to non-inflamed tissue or quiescent disease; furthermore, treatment responders demonstrate decreased Gzmb expression while remaining elevated for non-responders [12,14,15]. Building on this idea, our group previously generated ^68^Ga-NOTA-GZP, a novel peptide-based PET tracer that allows for non-invasive mapping and quantification of Gzmb activity [12,16,17]. We demonstrated that Gzmb PET could detect intestinal inflammation at a single imaging timepoint with a single therapeutic intervention [12]. While these prior studies established the feasibility of Gzmb PET for detecting inflammation, they did not characterize how Gzmb PET signals evolve dynamically over the course of treatment, nor did they compare responses across therapies with different mechanisms of action or provide mechanistic insight into the cellular basis of imaging findings. Here, we substantially advance this work by performing the first longitudinal, multi-timepoint Gzmb PET/CT imaging study across three mechanistically distinct, clinically relevant therapies (anti-TNF antibody, prednisolone, and anti-IL-23 antibody) in a DSS-induced colitis model. In this study, we evaluated Gzmb levels via serial Gzmb PET/CT imaging across three treatment approaches (using ^68^Ga-NOTA-GZP) in IL-10^−/−^ mice administered a dextran sulfate sodium (DSS) murine colitis model for two weeks. These include clinically relevant modalities (anti-TNF antibody, prednisolone, and anti-IL-23 antibody) that have different mechanisms of action. We hypothesized that Gzmb PET should reflect dynamic treatment efficacy correlating with underlying mechanism of action. To further understand this complex dynamic, we performed transcriptomics of the sites of interest and used a dataset of single-cell RNA sequencing from colitis mice to explore how each source can be impacted in cytotoxic inflammation. The broader goal of this work is further the development of Gzmb PET imaging as component of a precision medicine approach to therapeutic response monitoring in IBD for improved patient management.

## 2. Results

**Scheme 1:**
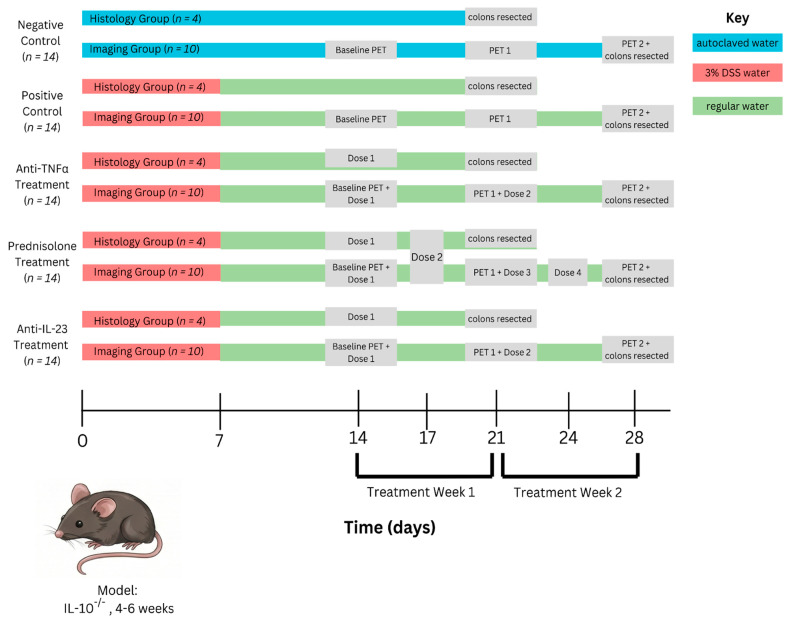
**Experimental timeline and study design.** Schematic overview of the experimental protocol. IL-10^−/−^ mice (4–6 weeks old) were divided into five groups (n = 14 each): negative control, positive control, anti-TNFα, prednisolone, and anti-IL-23 treatment. Colitis was induced with 3% DSS water for 7 days (days 0–7) in all groups except negative controls, which received autoclaved water throughout. Following DSS, mice were switched to regular water and treatments were initiated on day 14. Each group was subdivided into a histology cohort (n = 4) sacrificed at week 1 for early tissue analysis, and an imaging cohort (n = 10) undergoing serial ^68^Ga-NOTA-GZP PET/CT at baseline (day 14), week 1 (day 21), and week 2 (day 28) before sacrifice. Anti-TNFα and anti-IL-23 were administered weekly; prednisolone was given twice weekly.

The overall experimental design is illustrated in Scheme 1, including the timeline of DSS-induced colitis, treatment, and experimental endpoints. All DSS-exposed groups demonstrated clinical and histologic signs of colitis, while the negative controls remained asymptomatic. We first present the in vivo imaging data from Gzmb PET, followed by ex vivo approaches using IF and molecular analysis including bulk and single-cell RNA sequencing.

### 2.1. Granzyme B PET Imaging Reflects Disease Dynamics and Treatment Response

**Figure 1:**
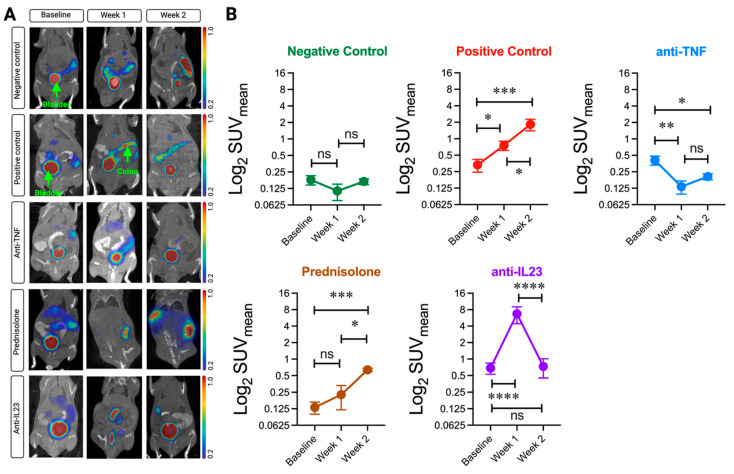
Granzyme B PET/CT imaging reveals distinct treatment response patterns in murine colitis. (**A**) Representative coronal PET/CT images obtained with 68Ga-NOTA-GZP at baseline (pre-treatment), week 1, and week 2 for each experimental group. Left panels show colon segmentation overlay on CT (red outline) with bladder indicated. Color scale represents SUV intensity (0–10 or as indicated). Negative control mice show consistently low colonic uptake. Positive control (untreated colitis) demonstrates progressive increase in tracer uptake over time. Anti-TNF-treated mice exhibit marked reduction in signal by week 1, maintained at week 2. Prednisolone-treated mice show persistent colonic uptake through week 2. Anti-IL-23-treated mice display striking elevation at week 1 followed by reduction at week 2. (**B**) Quantification of colonic tracer uptake expressed as Log_2_ SUV_mean_ for each treatment group across timepoints. Data are presented as mean ± SEM (n = 8–10 per group). Statistical comparisons between timepoints within each group are shown. * *p* < 0.05, ** *p* < 0.01, *** *p* < 0.001, **** *p* < 0.0001; ns, not significant (repeated measures ANOVA with post hoc analysis).

**Figure 2:**
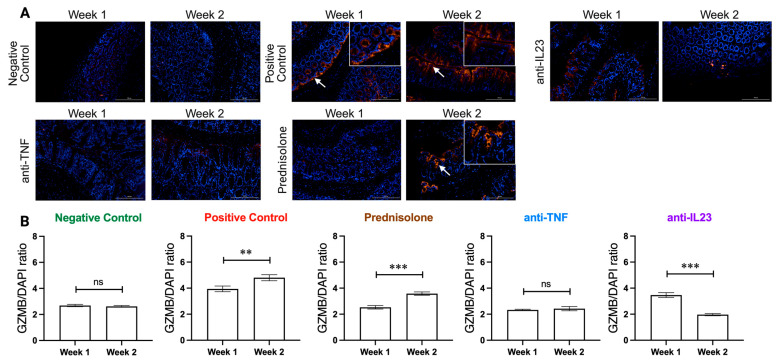
Immunofluorescence validation of granzyme B expression in colonic tissue. (**A**) Representative immunofluorescence images of colon sections stained for granzyme B (Gzmb, orange, Alexa Fluor 555) with nuclear counterstain DAPI (blue) at week 1 and week 2 of treatment for each experimental group. White arrowheads indicate representative Gzmb-positive regions. Insets show higher magnification of selected regions. Negative control shows minimal Gzmb signal at both timepoints. Positive control demonstrates progressive accumulation of Gzmb-positive cells in the lamina propria from week 1 to week 2. Anti-TNFα-treated tissue exhibits consistently low Gzmb expression similar to negative controls. Prednisolone-treated sections show low Gzmb at week 1 but increased signal at week 2. Anti-IL-23-treated tissue displays elevated Gzmb at week 1 with marked reduction by week 2. Scale bars = 200 μm. (**B**) Quantification of Gzmb expression calculated as the ratio of Gzmb fluorescence intensity to DAPI signal for each treatment group at week 1 and week 2. Data are presented as mean ± SEM (n = 4–6 per group per timepoint). ** *p* < 0.01, *** *p* < 0.001; ns, not significant (unpaired *t*-test or two-way ANOVA with post hoc analysis). Immunofluorescence findings corroborate PET imaging results, confirming granzyme B as a reliable tissue biomarker of treatment response.

In this section, we performed serial PET/CT imaging with ^68^Ga-NOTA-GZP to evaluate different patterns of colonic tracer uptake across experimental group (Figure 1). At the endpoint of the study, negative control IL-10^−/−^ mice showed consistently low intestinal uptake of ^68^Ga-NOTA-GZP (SUV_mean_ range of ~0.10–0.40, Figure 2). Positive control mice (IL10^−/−^ DSS colitis, untreated) exhibited a significant increase in uptake of the tracer. The SUV_mean_ from the colon in the positive control group increased from 0.33 ± 0.17 (baseline) to 0.76 ± 0.35 (week 1) and 1.83 ± 0.97 (week 2) (Figure 2A,B). This trend follows the expected progression of inflammation over time (baseline vs. week 2 *p* < 0.01). The PET imaging made evident that the positive controls at week 2 exhibited considerable and diffuse ^68^Ga-NOTA-GZP uptake across the colon, indicating the presence of active and ongoing inflammation (Figure 2A,B). In the anti-TNF treatment group, the Gzmb PET signal decreased significantly following therapy. The anti-TNF group exhibited elevated colonic uptake similar to positive controls at baseline (after DSS and before treatment), with an SUV_mean_ of 0.40 ± 0.21. Following one dose of anti-TNF (week 1), a significant reduction in Gzmb PET signal was observed (SUV_mean_ 0.13 ± 0.07, *p* < 0.01), and a statistically significant reduction was observed at week 2 following the second dose (SUV_mean_ 0.2 ± 0.06, *p* < 0.05 compared to baseline). In fact, qualitatively, the PET images of the anti-TNF-treated mice at week 2 were indistinguishable from healthy control mice, with only faint background uptake observed in the colon. In this study, we observed that the prednisolone group exhibited a divergent profile. At baseline, SUV_mean_ was observed at 0.13 ± 0.09, and after one week of prednisolone, the PET signal had not decreased meaningfully; in fact, their SUV_mean_ at week 1 was 0.23 ± 0.21, which was slightly higher than baseline (Figure 2B). By the end of week 2, mice receiving prednisolone demonstrated yet more Gzmb tracer uptake (SUV_mean_ 0.63 ± 0.06). The week 2 SUV_mean_ was significantly higher than the baseline (*p* < 0.001) and also higher than week 1 (*p* < 0.05), although it remained lower than the positive control. Interestingly, in the anti-IL23 treatment group, the Gzmb PET signal showed a biphasic pattern. At baseline, these mice had moderate levels of colonic uptake of 0.68 ± 0.44. However, after one week of anti-IL23 treatment, instead of decreasing, SUV_mean_ increased to 6.70 ± 5.10 (Figure 2B), which is nearly a 10-fold increase from baseline (*p* < 0.001), and the largest uptake that we observed in any group/timepoint. Visually, the week 1 PET images in this group were quite striking as they showed intense hotspots of tracer within the colon (Figure 2A). By the two-week mark in the study, the anti-IL23 group’s PET signal decreased significantly (*p* < 0.001). The mean SUV for the anti-IL23 group was 0.73 ± 0.48 at week 2, which is approximately the baseline level and lower than week 1 (*p* < 0.001). In fact, at week 2, uptake in anti-IL23 mice was similar to baseline (0.73 vs. 0.68, *p* > 0.05).

### 2.2. Immunofluorescence Confirms Granzyme B as a Biomarker of Treatment Response

Next, we tried to confirm that the PET signal changes reflected differences in Gzmb levels in colon tissues. We performed Gzmb immunofluorescence (IF) staining on colon sections from each group and timepoint. Figure 2A shows representative IF images for each animal group after week 1 and week 2 of treatment. Quantitative image analysis of IF images showing the Gzmb/DAPI fluorescence ratio is shown in Figure 2B. Overall, the IF results corresponded with the imaging results, confirming Gzmb as a biomarker of immune activity. In animals that served as a negative control, there was minimal staining for Gzmb at both timepoints. We noted only sporadic Gzmb+ cells in the lamina propria, which is similar to the background (and probably reflects a minimal presence of cytotoxic cells at baseline in the normal colon). The Gzmb/DAPI ratio of negative control mice was approximately 2.7, which was lower than any of the other groups. For the positive control colitic mice, we observed high expression of Gzmb and this was even more dramatically increased at the 2-week timepoint. At week 1 post-DSS, positive control colons had an abundance of Gzmb-positive cells scattered in both the inflamed mucosa and submucosa (Figure 2A). These cells appear as red clusters correlated with infiltrating immune cells in the lamina propria and occasionally appear slightly deeper into the tissue in crypt. By week 2, the density of Gzmb+ cells was even greater and we noted that large areas of the mucosa were filled with red fluorescence, which indicated that active inflammation remained (2 weeks post-injury). The quantification of Gzmb staining demonstrated that the Gzmb/DAPI ratio for the positive controls increased from 3.9 ± 3.1 at 1 week to 4.8 ± 3.3 at week 2 (*p* < 0.01), indicating an ongoing accumulation of Gzmb-expressing cells following DSS-induced colitis (Figure 2B). This indicates that in untreated disease, Gzmb expression worsens along with inflammation and coincides with our previous data from humans who have inflammatory bowel disease (IBD), showing that Gzmb is higher in tissues that have an active IBD phase compared to quiescent IBD. In the anti-TNF group, Gzmb expression was markedly lower at both timepoints compared to positive controls. Following one week of anti-TNF treatment, IF analysis exhibited only isolated Gzmb-positive cells in the colonic tissues (Figure 2A), and the staining appeared to be at a background level by 2 weeks, similar to the negative controls. The few Gzmb^+^ cells noted were small, isolated, and commonly located near lymphoid aggregates in the submucosa but far removed from the extensive staining present in untreated colitis. The Gzmb/DAPI ratios in anti-TNF mice were 2.3 ± 0.7 at week 1 and 2.4 ± 2.3 at week 2, both significantly lower than positive controls (*p* < 0.001), while there was no significant difference at week 2 compared to negative controls (Figure 2B). The prednisolone-treated mice exhibited a more complex profile of IF. After over a week of steroid treatment, Gzmb staining was relatively low compared to the positive control group (week 1, 2.5 vs. 3.9). However, after 2 weeks of prednisolone treatment, there was a substantial increase in Gzmb-positive cells in the IF (from 2.5 ± 1.8 at week 1 to 3.6 ± 1.8). Patches of mucosa displayed orange fluorescence, indicating that Gzmb-expressing cytotoxic cells had increased in abundance once again (Figure 2A, week 2 prednisolone), which this reflects the PET SUV increase at week 2. Finally, the anti-IL23 group’s IF results paralleled their overall PET pattern. At 1 week of anti-IL23, we found surprisingly high levels of Gzmb staining (Figure 2A). In fact, the intensity and distribution of Gzmb+ cells in the week 1 anti-IL23 group was similar to positive controls at 1 week (3.5 ± 2.6 vs. 3.9 ± 3.1, *p* > 0.05). There were clusters of Gzmb-expressing cells particularly in the lamina propria of the distal colon. The Gzmb/DAPI ratio at week 1 for anti-IL23 was 3.5 ± 2.6, which was significantly higher than the anti-TNF or prednisolone groups (*p* < 0.001). This clearly confirms that IL23 blockade initially coincided with an augmentation of Gzmb-expressing cell activity. Then, by week 2, the anti-IL23 group underwent a significant reduction in Gzmb staining. The week 2 anti-IL23 colon sections only had sparse Gzmb+ cells remaining (Figure 2A), just like the anti-TNF group. The Gzmb/DAPI ratio had fallen to 1.9 ± 1.2 at week 2 which was a significant reduction from week 1 (*p* < 0.001) and significantly lower than the week 2 positive controls (*p* < 0.001; Figure 2B). Thus, the IF data confirmed a transient surge in Gzmb expression with anti-IL23 therapy, followed by a later suppression. Mechanistically, this may relate to the role of IL-23 in maintaining certain regulatory feedback loops.

### 2.3. Transcriptional Profiling Identifies the Immune Mechanisms of Imaging Changes

**Figure 3:**
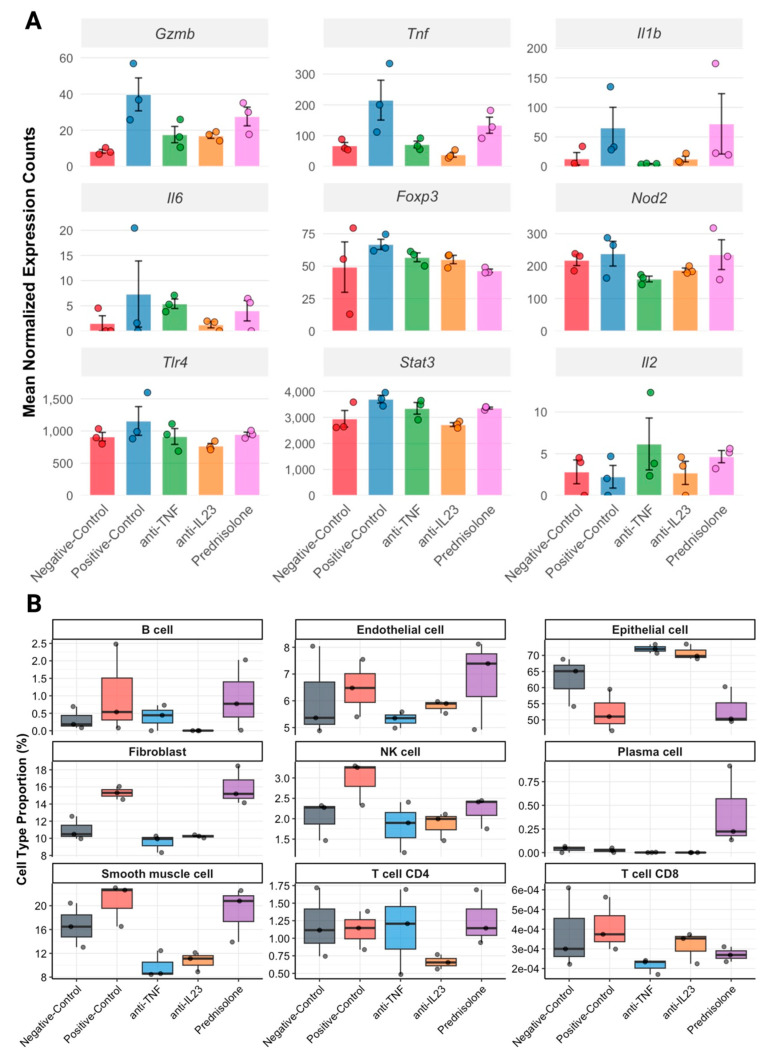
Bulk RNA sequencing analysis of inflammatory gene expression and cell-type deconvolution in colitis treatment groups. (**A**) Mean normalized expression counts of key inflammatory and immune-related genes across treatment groups. Genes analyzed include Gzmb, Tnf, Il1b, Il6, Foxp3, Nod2, Tlr4, Stat3, and Il2. The positive control (DSS colitis, untreated) demonstrates elevated expression of Gzmb and Tnf compared to the negative control, with treatment groups showing variable responses. Data are presented as mean ± SEM with individual data points shown (n = 3–4 per group). (**B**) Cell-type proportion estimates derived from cell deconvolution analysis using a murine DSS-induced colitis scRNA-seq reference dataset. Box plots display estimated proportions of B cells, endothelial cells, epithelial cells, fibroblasts, NK cells, plasma cells, smooth muscle cells, CD4+ T cells, and CD8+ T cells across treatment groups. Notable findings include reduced epithelial cell proportions in positive controls reflecting colitis-associated epithelial damage, elevated NK cell and fibroblast proportions in positive controls consistent with active inflammation and stromal remodeling, and variable restoration patterns across treatment groups.

In this step, we tried to mechanistically elucidate the patterns of Gzmb PET signals we observed and analyze the changes in the gene expression profiles in colon tissues using bulk RNA-seq, which we also further correlated with single-cell RNA-seq datasets to explore the relationship between Gzmb expression and cellular changes in different settings of IBD and treatment regimens. The bulk RNA-seq differentially expressed genes supported our imaging and histology findings with a focus on key inflammatory mediators targeted by each of the therapies at the end of week 2 post-treatment. Among the pro-inflammatory genes that were differentially expressed, Gzmb was a major gene that was regulated (Figure 3A). In comparing untreated colitis to healthy tissue, Gzmb was upregulated >5-fold, confirming it as a hallmark of active disease consistent with human data (upregulation ~3 fold in active UC). In mice treated with anti-TNF, Gzmb expression significantly decreased to near baseline levels by week 2 (but was still higher than the negative control group), showing a greater reduction (greater than 4-fold lower than untreated colitis) than either Tnf or Il1b that changed about 2-fold with anti-TNF (Figure 2A). When observed in prednisolone-treated samples, Gzmb did not show a dramatic decrease, as observed in anti-TNF, which was consistent with the PET/IF showing that cytotoxic activity was still present through week 2. In addition, prednisolone was associated with modest downregulation of transcripts in some inflammatory cytokines (Il6 and Ifng were down modestly and Tnf was down ~1.5-fold) similar to Gzmb, suggesting that prednisolone may not completely restore control over cytotoxic lymphocytes. In the anti-IL23-treated colons, the Gzmb expression pattern was similar to anti-TNF at week 2. Anti-IL-23 largely impacted Il1b and Tnf by the end of week 2, as well as Gzmb expression. Overall, among the pro-inflammatory markers, Gzmb and Tnf showed the most meaningful dynamic changes across different treatment regimens. Notably, the expression pattern of Gzmb closely mirrored that of Tnf, suggesting that Gzmb is as effective as Tnf for evaluating disease status.

Figure 3B presents cell-type proportion estimates derived from cell deconvolution analysis using a scRNA-seq murine DSS-induced colitis model as the reference. The analysis revealed distinct patterns of cellular composition across treatment groups. NK cell abundance was significantly elevated in the positive control group compared to the negative control, consistent with active cytotoxic inflammation. All therapeutic interventions (anti-TNF, anti-IL-23, and prednisolone) were associated with reduced NK cell proportions relative to the positive control, though the magnitude of reduction varied among treatments. Similarly, CD8^+^ T cell proportions showed treatment-associated changes, with anti-IL23 and prednisolone groups displaying lower estimated abundances. Epithelial cell proportions were markedly reduced in the positive control group compared to the negative control, reflecting epithelial damage characteristic of colitis. Treatment groups, particularly anti-IL23, showed partial restoration of epithelial cell proportions toward baseline levels. Conversely, fibroblast and smooth muscle cell proportions were elevated in the positive control group, suggesting stromal expansion associated with inflammation and tissue remodeling. These proportions were reduced in the anti-TNF and anti-IL23 treatment groups, while prednisolone-treated samples maintained relatively elevated smooth muscle cell proportions. Endothelial cell proportions appeared highest in the prednisolone group. CD4^+^ T cell proportions varied across groups, with prednisolone treatment associated with lower estimated abundance. B cell and plasma cell proportions remained relatively low across all conditions.

**Figure 4:**
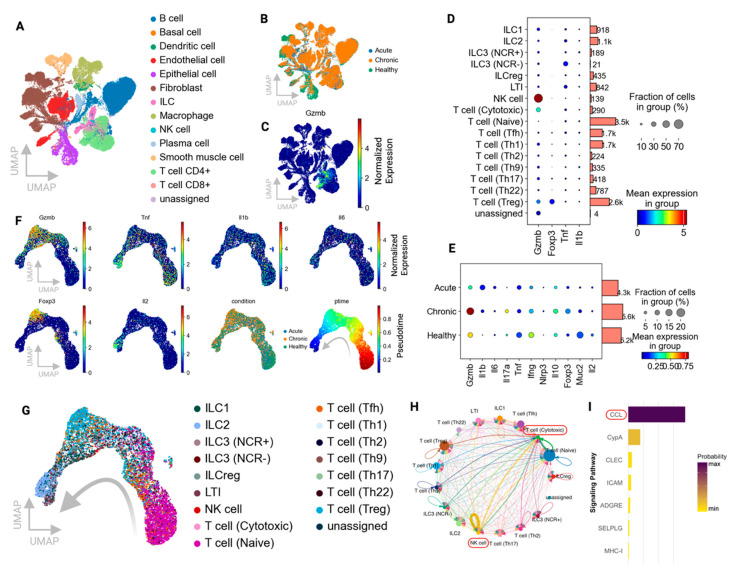
Single-cell RNA sequencing analysis of Gzmb expression and immune cell dynamics in murine DSS-induced colitis. (**A**) UMAP projection of all cell types identified in the scRNA-seq dataset (GSE264408). (**B**) UMAP visualization colored by disease condition: healthy, acute colitis, and chronic colitis. (**C**) UMAP displaying normalized Gzmb expression across all cells. (**D**) Dot plot showing Gzmb, Foxp3, Tnf, and Il1b expression across immune cell subsets; dot size represents the fraction of cells expressing each gene, and color intensity indicates mean expression level. (**E**) Dot plot comparing expression of inflammatory markers across disease conditions; Gzmb demonstrates the most pronounced elevation in chronic colitis relative to acute colitis and healthy tissue. (**F**) Feature plots showing expression of key inflammatory genes (Gzmb, Tnf, Il1b, Il6, Foxp3, and Il2), disease condition distribution, and pseudotime trajectory on UMAP; lower pseudotime values correspond to more activated effector states. (**G**) UMAP of immune cell subsets showing detailed annotation of ILC and T cell populations along the pseudotime trajectory. (**H**) CellChat interaction network illustrating cell–cell communication among immune populations; edge thickness represents interaction strength, with prominent interactions between NK cells and cytotoxic T cells. (**I**) Analysis of signaling pathway probability scores, identifying CCL signaling as the predominant pathway mediating immune cell interactions.

To further explore the relationship between Gzmb expression, disease status, and the cellular sources of Gzmb in colitis, we analyzed publicly available scRNA-seq data from a murine DSS-induced colitis model encompassing three disease stages: healthy, acute colitis, and chronic colitis (GSE264408). Figure 4 presents the comprehensive analysis. After quality control and preprocessing, cells were clustered based on gene expression features. Figure 4A displays the UMAP projection of all cell types identified in the dataset, while Figure 4B shows the distribution of cells across disease conditions (healthy, acute, and chronic colitis). Figure 4C illustrates the normalized Gzmb expression across all cells, revealing distinct localization of Gzmb-expressing cells within specific clusters. Among all immune cell subsets, NK cells exhibited the highest proportion of Gzmb-positive cells with the strongest mean expression levels compared to other cell types (Figure 4D). Cytotoxic T cells also demonstrated notable Gzmb expression, though at lower levels than NK cells. Importantly, Figure 4E demonstrates that Gzmb expression was most significantly elevated during the chronic phase of disease compared to both acute colitis and healthy conditions. Notably, acute colitis showed lower Gzmb expression than healthy controls. Among all inflammatory markers examined (including Il1b, Il6, Il17a, Tnf, Ifng, Nlrp3, Il10, Foxp3, Muc2, and Il2), Gzmb displayed the most robust dynamic changes, distinguishing chronic colitis from acute colitis and healthy tissue, while other markers showed less consistent patterns across disease states (Figure 4E). To evaluate the differentiation trajectory of immune cells, we extracted immune cell populations and performed ScTour analysis to order cells based on their differentiation state, assigning pseudotime values where a higher pseudotime represents cells closer to a naïve state and more inactive status and a lower pseudotime indicates more active cells responding to inflammation. Strikingly, Gzmb expression was significantly upregulated as cells progressed toward lower pseudotime values (more activate states), a dynamic pattern observed only in select inflammatory markers such as Il2 and Foxp3 (Figure 4F). To validate the analysis, we visualized immune cell subtype clustering (Figure 4G), confirming that naïve T cells predominated at higher pseudotime values while more activated populations, including ILC1, ILC2, and ILC3 subsets, occupied lower pseudotime regions. Finally, we performed CellChat analysis to examine cell–cell communication networks among immune populations. Based on the interaction weight and number, NK cells demonstrated the highest interaction frequency with cytotoxic T cells (Figure 4H), supporting the hypothesis that crosstalk between these two cell types may drive Gzmb expression in NK cells. Notably, the directionality of interactions was predominantly from cytotoxic T cells toward NK cells. Pathway analysis revealed that the CCL (chemokine ligand) signaling pathway was the most probable pathway mediating NK cell activation and subsequent Gzmb expression (Figure 4I).

## 3. Discussion

This study demonstrates that Gzmb-targeted PET imaging provides a dynamic, non-invasive window into intestinal immune activity and treatment response in experimental colitis. Using serial ^68^Ga-NOTA-GZP PET/CT imaging across three mechanistically distinct therapies, we uncovered distinct temporal patterns of the Gzmb signal that reflect the underlying immunomodulatory actions of each treatment. These imaging findings were corroborated by immunofluorescence staining, histopathology, and transcriptomic profiling, establishing Gzmb PET as a sensitive and specific biomarker of cytotoxic immune activity in the gastrointestinal tract. Importantly, the close agreement between PET signal and tissue-level Gzmb protein expression across all treatment groups—including conditions where the PET signal dissociated from overall histological inflammation—provides strong evidence for target-specific tracer uptake in vivo [12,16].

A central finding of this study is that each therapeutic intervention produced a distinctive Gzmb PET signature that aligned with its known mechanism of action. Anti-TNF therapy resulted in rapid and sustained suppression of the Gzmb signal, with near normalization by week 2 of treatment. This pattern is consistent with the established effects of TNF neutralization, which include a reduction in the inflammatory cytokine cascade, induction of apoptosis in activated immune cells, and suppression of lymphocyte recruitment to inflamed tissues [18,19,20]. The swift response observed with anti-TNF mirrors clinical scenarios where this class of biologics can induce remission and mucosal healing within weeks of initiation and suggests that Gzmb PET could serve as an early indicator of anti-TNF response. In contrast, prednisolone treatment yielded a suboptimal response characterized by a persistently elevated, and even increasing, Gzmb PET signal through week 2. This finding was initially counterintuitive given the potent anti-inflammatory properties of corticosteroids. However, steroids primarily act by inhibiting the transcription of inflammatory mediators, such as IL-1, TNF, and chemokines, and by reducing neutrophilic inflammation [21,22]. Importantly, corticosteroids do not directly target cytotoxic lymphocyte populations and may have limited efficacy in suppressing activated CD8^+^ T cells and NK cells—the principal sources of Gzmb. Our data align with clinical observations that, while steroids effectively induce symptomatic remission, they frequently fail to achieve complete mucosal healing or sustained remission, and steroid-dependent patients often experience relapse upon tapering [23]. The elevated Gzmb signal in steroid-treated mice may thus represent residual cytotoxic immune activity that predisposes to disease recurrence, a phenomenon that Gzmb PET could potentially identify in clinical practice. Perhaps the most striking observation was the biphasic response pattern in the anti-IL-23 treatment group. Following a single dose of anti-IL-23, the Gzmb PET signal increased nearly 10-fold at week 1—the highest uptake observed in any group—before declining significantly by week 2 to levels comparable to the baseline. This paradoxical early elevation likely reflects the complex immunoregulatory role of IL-23 in intestinal inflammation. IL-23 is essential for maintaining Th17 cell populations and promoting IL-17/IL-22 production, and its blockade effectively suppresses this pathway [24,25]. However, IL-23 neutralization does not directly inhibit CD8+ T cells, which may continue to be activated by alternative cytokines such as IL-15, IL-21, or IL-12 in the absence of Th17-mediated counter-regulation [24,26,27]. Studies on checkpoint inhibitor-induced colitis models have demonstrated that while IL-23 blockade reduces CD4^+^ T cell-driven inflammation, residual disease may persist due to ongoing CD8+ T cell cytotoxicity with preserved IFNγ and granzyme B production [24,28]. Our findings suggest that early IL-23 blockade may transiently disinhibit or even expand cytotoxic lymphocyte populations before the overall inflammatory milieu is sufficiently dampened to reduce their activity. By week 2, sustained IL-23 blockade likely interrupts the tissue damage–antigen presentation–immune activation loop, leading to reduced CD8^+^ T cell activation and decreased Gzmb production. This biphasic pattern has important clinical implications. In practice, patients initiating IL-23 inhibitor therapy may experience transient symptom flares or apparent non-response before eventual improvement—a phenomenon that could lead to premature discontinuation of effective therapy. Gzmb PET imaging could potentially distinguish such delayed responders from true non-responders by revealing the temporal evolution of cytotoxic immune activity and identifying the inflection point at which inflammation begins to resolve.

An important observation from our study is the dissociation between Gzmb PET signal and histopathological scores in certain treatment contexts. In anti-TNFα-treated mice, the PET signal showed near-normalization by week 2, whereas histology still revealed residual chronic inflammatory changes. This suggests that Gzmb PET captures the functional resolution of active cytotoxic immunity—the cessation of ongoing immune-mediated tissue injury—even as structural healing continues. Conversely, prednisolone-treated mice demonstrated improved histology scores despite an elevated Gzmb PET signal, indicating that while gross tissue architecture may be improving, active cytotoxic immune cells remain present and capable of perpetuating damage. This distinction has profound clinical relevance. Endoscopic assessment of mucosal healing remains the cornerstone of IBD monitoring and provides critical information about disease status. However, endoscopy primarily evaluates structural appearance that may not fully reflect underlying immune activity. A mucosa that appears healed endoscopically could harbor active cytotoxic lymphocytes—a “smoldering” inflammatory state that predisposes to relapse. Gzmb PET offers a complementary assessment of immune function that could identify patients at risk for disease recurrence despite apparent mucosal healing, thereby informing decisions about treatment intensification or maintenance therapy.

Our transcriptomic analyses, including bulk RNA sequencing with cell deconvolution and integration with single-cell RNA sequencing datasets, provided mechanistic insight into the cellular basis of imaging findings. Cell deconvolution analysis revealed that NK cell proportions were elevated in untreated colitis and reduced with effective therapy, paralleling Gzmb expression patterns. Single-cell analysis of the GSE264408 dataset confirmed that NK cells exhibited the highest proportion of Gzmb-positive cells with the strongest mean expression levels among immune populations, followed by cytotoxic T cells. Importantly, Gzmb expression was most significantly elevated during chronic colitis compared to both acute colitis and healthy tissue, with acute colitis paradoxically showing lower Gzmb expression than healthy controls. This pattern suggests that Gzmb is a marker of established, actively cytotoxic inflammation characteristic of chronic disease rather than early acute inflammatory responses. Among all inflammatory markers examined, Gzmb demonstrated the most robust dynamic changes distinguishing chronic from acute colitis, supporting its utility as a specific biomarker of cytotoxic immune activation. Pseudotime trajectory analysis revealed that Gzmb expression increased as immune cells transitioned toward more activated effector states, a pattern observed only in select markers such as Il2 and Foxp3. CellChat analysis of cell–cell communication networks identified prominent interactions between NK cells and cytotoxic T cells, with the directionality predominantly from cytotoxic T cells toward NK cells. The CCL (chemokine) signaling pathway emerged as the most probable mediator of these interactions, suggesting that chemokine-mediated crosstalk between T cells and NK cells amplifies cytotoxic activity in the inflamed colon. These findings provide a mechanistic framework for understanding how Gzmb-producing cells are recruited and activated in colitis and how effective therapies may disrupt these communication networks.

Our findings reinforce the dual significance of Gzmb in IBD pathophysiology. Beyond serving as a biomarker of cytotoxic immune activity, Gzmb functions as an active effector of tissue injury. Extracellular Gzmb contributes to inflammation through the degradation of extracellular matrix components, activation of matrix metalloproteinases, and promotion of epithelial barrier dysfunction [29]. Thus, imaging Gzmb activity provides not merely a surrogate measure of immune cell presence but a direct visualization of an ongoing pathogenic process. The ability to monitor and modulate Gzmb-expressing cell populations may therefore have therapeutic implications beyond disease monitoring.

While these preclinical findings are promising, the translational path from murine colitis to human IBD management requires careful consideration of the fundamental differences between these settings. The DSS model produces synchronized, acute-to-subacute inflammation in young mice over weeks, whereas human IBD is a chronic, relapsing–remitting disease that progresses over months to decades and involves tissue remodeling, fibrosis, and structural complications such as strictures and fistulae that are absent from our model. Nevertheless, the biological principle underlying our findings—that Gzmb activity reflects ongoing cytotoxic immune-mediated tissue injury and responds dynamically to effective immunomodulation—is supported by human data showing elevated mucosal Gzmb in active IBD that decreases with successful treatment [12,14,15]. We therefore cautiously advance the translational potential of Gzmb PET imaging for IBD management. We envision Gzmb PET as complementary to existing monitoring strategies, with three potential clinical applications: (1) confirmation of active cytotoxic inflammation when symptoms are ambiguous, endoscopy is contraindicated, or disease is located in segments not accessible by colonoscopy; (2) early assessment of treatment response within weeks of therapy initiation, enabling timely decisions about treatment continuation or modification; and (3) prediction of relapse risk based on residual subclinical immune activity. Gzmb PET provides information that is complementary to existing biomarkers. Fecal calprotectin primarily reflects neutrophilic inflammation and cannot distinguish cytotoxic lymphocyte-driven disease. CRP lacks specificity and cannot localize inflammation. Gzmb PET specifically reports on T cell and NK cell activity, potentially identifying patients who would benefit most from therapies targeting these populations. Although direct head-to-head comparisons with FDG PET and fecal calprotectin were not performed in this study, the mechanistic specificity of 68Ga-NOTA-GZP for cytotoxic immune cells—supported by our single-cell transcriptomic data—suggests that Gzmb PET captures a distinct immunological compartment not reflected by these existing modalities. Furthermore, Gzmb PET can assess the entire bowel, including small intestinal segments inaccessible to colonoscopy—a significant advantage for Crohn’s disease patients with proximal disease. The combination of Gzmb PET with other molecular imaging approaches or biomarkers could provide a comprehensive immune profile of IBD lesions. For example, a patient with a high Gzmb PET signal but low calprotectin might have predominantly cytotoxic lymphocyte-driven inflammation, whereas the reverse pattern might suggest neutrophil-dominated disease, each potentially requiring different therapeutic strategies.

Several limitations warrant acknowledgment. First, the IL-10^−/−^ DSS model, while combining chronic immune dysregulation with acute injury, does not fully recapitulate the complexity of human IBD. The synchronized disease onset, compressed timescale (weeks vs. months–years), absence of chronic tissue remodeling (fibrosis and strictures), and lack of the relapsing–remitting pattern characteristic of human IBD all limit direct extrapolation of our quantitative findings. Furthermore, treatment response in humans is assessed over considerably longer intervals, and the specific temporal patterns observed (e.g., the biphasic anti-IL-23 response over 2 weeks) may manifest over different timescales clinically. Additionally, because Gzmb is a shared effector molecule of cytotoxic lymphocytes in both inflammatory and antitumor immune responses, an elevated Gzmb PET signal is not inherently specific to colitis. In our preclinical model, this was not a confounding factor: the young age of the mice (4–6 weeks), short experimental duration (~4 weeks), and absence of dysplasia or neoplasia on systematic histological examination preclude cancer-associated Gzmb activity. However, in clinical application—particularly in IBD patients with longstanding disease who carry elevated risk for colitis-associated colorectal cancer—antitumor immune responses could contribute to Gzmb PET signals and would need to be distinguished through endoscopic correlation and analysis of uptake patterns. Second, while our overall sample size was adequate, subgroup sizes for molecular analyses were modest, potentially limiting statistical power for some comparisons. Third, although ^68^Ga-NOTA-GZP was effective in mice and the peptide sequence is cross-reactive with human Gzmb, clinical translation will require formal safety, dosimetry, and optimization studies. Fourth, while the specificity of 68Ga-NOTA-GZP for Gzmb has been established through in vitro binding assays and prior imaging studies [12,16], in vivo blocking or competitive inhibition studies were not performed in the current colitis model; dedicated blocking experiments in future studies would provide additional confirmation of in vivo specificity. Fifth, we did not directly compare Gzmb PET with FDG PET, MR enterography, or fecal calprotectin; head-to-head studies are needed to empirically define the added value of Gzmb-specific imaging over existing modalities. Sixth, while we propose Gzmb PET as a complementary tool for disease monitoring, PET/CT carries its own practical constraints for clinical use, including ionizing radiation exposure, cost, and the need for access to 68Ga generators and PET/CT facilities, which would limit its use to select clinical scenarios rather than routine serial surveillance.

Building on these findings, several future directions merit pursuit. Clinical studies evaluating Gzmb PET in IBD patients initiating new therapies could determine whether early imaging (2–4 weeks post-treatment) predicts endoscopic remission or long-term outcomes. A combination immuno-PET approaches—imaging Gzmb alongside T cell activation markers or trafficking molecules—could provide multidimensional immune profiling. Beyond IBD, Gzmb PET may have applications in other conditions involving cytotoxic lymphocytes, including graft-versus-host disease, immune checkpoint inhibitor-induced toxicities, and organ transplant rejection. Finally, the integration of Gzmb PET quantification with machine learning algorithms could enable automated prediction of treatment response.

## 4. Materials and Methods

### 4.1. Animals and Experimental Groups

All procedures involving animal subjects were authorized by the Institutional Animal Care and Use Committee and followed ARRIVE (Animal Research: Reporting of In Vivo Experiment) guidelines. Male C57BL/6 IL-10^−/−^ mice (Jackson Laboratories, 4–6 weeks old) were utilized as this strain develops chronic colitis spontaneously due to dysregulated immune responses [30]. A total of 70 mice were acclimated and then randomly assigned to five groups of 14 each: (1) negative control, (2) positive control, (3) anti-TNF treatment, (4) prednisolone treatment, and (5) anti-IL-23 treatment.

To induce colitis, mice in all groups except for the negative control group were administered dextran sulfate sodium (DSS) in their drinking water ad libitum. Mice were given a 3% DSS solution (36–50 kDa, MP Biomedicals) in autoclaved water for 7 days, after which mice were returned to regular water for the remainder of the study period. In this study, exposure to DSS causes acute colonic injury and local inflammation; however, in IL-10^−/−^ mice, this results in progression to a chronic colitic state [31]. The negative control group received autoclaved water without DSS; therefore, they never developed colitis throughout the course of the study. All other groups (positive control and treatment groups) were subject to the same first week of 3% DSS to induce colitis, then switched to normal drinking water, to initiate the recovery phase or chronic phase of inflammation followed by treatment. Mice were evaluated at least three times weekly for clinical signs of colitis, including weight loss, stool consistency (diarrhea), evidence of fecal occult or gross blood, and overall appearance/activity. Disease activity index was measured as a composite of percent weight loss, stool consistency, and bleeding to assess colitis induction and to determine treatment effects. A schematic overview of the experimental timeline and group assignments is included in Scheme 1. Animals underwent a baseline PET scan two weeks after DSS initiation (i.e., one week after DSS withdrawal and immediately before treatment initiation), and after treatment was initiated, the animals received follow-up PET imaging once and then again at two weeks post-initiation of treatment, one week apart.

Group Descriptions: The negative control group (Group 1) comprised IL-10^−/−^ mice that did not receive DSS, to account for any background Gzmb signals intrinsic to the IL-10^−/−^ model. The positive control group (Group 2) consisted of IL-10^−/−^ mice with DSS-induced colitis who did not receive treatment, thus indicative of ongoing inflammation. The treatment groups were Group 3: anti-TNF; Group 4: prednisolone; and Group 5: anti-IL-23. Each group had an initial 14 mouse group size; however, only 10 mice per group underwent the full imaging experiments (baseline, week 1, and week 2 scans) before euthanasia for tissue collection after the last scan (2 weeks of treatment). The remaining four mice in each group were assigned to early endpoint histology after one week of treatment (to allow for tissue to evaluate early treatment efficacy without a subsequent imaging session). This design facilitated paired imaging and histology across two intervals (1 and 2 weeks after treatment) while limiting stress related to imaging and minimizing stress in the subset of early sacrifice. All animals were humanely euthanized per American Veterinary Medical Association guidelines by CO_2_ asphyxiation using a gradual-fill method to minimize distress.

### 4.2. Treatment Administration

Following DSS-mediated colitis, the treatment groups began receiving their respective therapies one week after DSS withdrawal. Therapeutics were administered via intraperitoneal (i.p.) injection at doses informed from the previous literature and pilot work. Antitumor necrosis factor α (anti-TNFα) therapy involved a monoclonal antibody against mouse TNFα (clone XT3.11, Bio X Cell #BE0058) given at 100 µg. Anti-TNFα was given once weekly via i.p. injection [32]. The present dosing was informed from studies demonstrating effective TNFα neutralization in disease models, including colitis in IL-10^−/−^ mice [32]. Prednisolone (Sigma-Aldrich, St. Louis, MO, USA) was administered at a dose of 10 mg/kg body weight (bw) via i.p. injection twice weekly (every 3–4 days) [23]. The chosen glucocorticoid dosing aimed to represent moderate steroid treatment for active colitis considering the desired efficacy and toxicity. Anti-IL-23 therapy utilized a monoclonal antibody against the p19 subunit of IL-23 (clone G23-8, Bio X Cell #BE0313) at a dose of 100 µg, given once weekly via i.p. injection in a similar manner as anti-TNFα therapy. Blockade of IL-23 p19 in animal models of IBD has been shown to reduce the severity of colitis, correlating with inactivity of Th17/Th1 responses [24], and the present dosing was informed from studies utilizing effective doses in preclinical inflammatory models. All treatment interventions were diluted in sterile saline for administration (final volume ~200 µL). The positive control experimental group received sham injections (saline vehicle in the same volume/schedule) to control for any stress effects of the injection. The negative control group received no injections (or saline only), as they were not subject to DSS-induced colitis. During the entire treatment period, mice were extensively monitored for any adverse effects of the therapies. In this regard, body weights were monitored every 2–3 days. All groups exposed to DSS initially lost weight (~5–15%), and while under treatment, the anti-TNFα- and anti-IL-23-treated groups exhibited weight stabilization or gain by week 2, whereas the prednisolone groups had more variable weight curves.

### 4.3. ^68^Ga-NOTA-GZP Radiotracer Synthesis

The Gzmb-targeted PET tracer ^68^Ga-NOTA-GZP was synthesized on-site according to previously described methods [12]. The peptide inhibitor sequence GZP (sequence: (β-Ala)-Gly-Gly-Gly-Ile-Glu-Phe-Asp-CHO, received with a NOTA chelator at N-terminus) was purchased (Peptides International) or synthesized as previously described [12]. Radiolabeling via Gallium-68 was performed using a ^68^Ge/^68^Ga generator (Eckert & Ziegler, Berlin, Germany) as the source of ^68^Ga. Briefly, ^68^GaCl_3_ (in 0.1 M HCl eluent from the generator) was mixed with 0.8 mg NOTA-GZP peptide in 1 M ammonium acetate buffer (pH ~4). Incubation at room temperature for 15 min allowed the chelation of ^68^Ga to the NOTA moiety (final pH of about 4). Upon incubation, the preparation was then purified via a C18 Sep-Pak cartridge (Waters, Milford, MA, USA) to isolate the radiolabeled compound from unbound, free ^68^Ga and impurities, as previously described in our prior study [12]. The cartridge was pre-conditioned and loaded with the reaction mixture. The radiolabeled product was retained on the cartridge and eluted with a small volume of ethanol or ethanol/water mixture, which was subsequently evaporated, or further diluted in saline for administration. The radiochemical purity of the final product was determined using instant thin-layer chromatography (iTLC) with silica gel strips developed in a suitable mobile phase (e.g., 0.1 M citrate buffer). Free ^68^Ga (in the form of Ga-citrate) was confirmed and remained at the origin or solvent front distinctly from the spot of the radiotracer. Similar to iTLC, radio-HPLC confirmed a radiochemical purity of >95% for all preparations of the tracer. The radiochemical yield of the labeling reaction was generally 60–70%. Specific activity was on the order of 5–15 MBq per µg peptide. Endotoxin testing and pH were performed on the final product prior to injections to ensure safety. Lastly, the tracer was formulated in sterile PBS or normal saline and filtered via a 0.22 µm filter and aliquoted in a sterile dose vial.

### 4.4. Small Animal PET/CT Imaging Procedures

Imaging Timepoints: Mice in the imaging cohorts (10 per group) were imaged at three timepoints: baseline (pre-treatment, which was approximately Day 14 from experiment start, or approximately a week after DSS), 1-week post-treatment, and 2 weeks post-treatment (end of study). Imaging was carried out using a small animal PET/CT scanner (TriFoil Imaging). For each imaging instance, mice were fasted for 4 h prior to tracer administration. Fasting prior to tracer administration was performed to reduce nonspecific abdominal uptake in the mice.

*Tracer injection*: Mice were then anesthetized with 2% isoflurane in 100% O_2_ and were held in a supine position on a warming pad for tracer administration. Approximately 5–8 MBq of ^68^Ga-NOTA-GZP (~135–220 microcuries (µCi) in 100–200 µL of sterile saline) was then injected intravenously via a lateral tail vein. (The mass of the peptide injected for the study was approximately 1–2 µg per mouse, which is well below the mass amount needed to induce saturation of Gzmb peptide-binding sites.) Activity delivered to each mouse was measured with a dose calibrator to determine the administered activity and enable standard uptake value (SUV) calculation. Following tracer injection, the anesthetized mice recovered prior to dosing the mice with the contrast agent during the uptake phase.

Uptake period and contrast agent: The tracer was allowed to circulate for 60 min to allow for blood clearance and binding of the target(s), which is supported with prior studies and pharmacokinetic optimization that show peak target vs. background contrast around 1-h post-injection. Then, 15 min prior to PET image acquisition, the mice were re-anesthetized (2% isoflurane) and administered an intrarectal dose of diatrizoate meglumine/diatrizoate sodium contrast (Gastrografin, Bracco Diagnostics). In detail, 200 µL of Gastrografin diluted in PBS (~1:1) was carefully infused through a flexible catheter placed into the rectum. This contrast agent enhances the visualization of the large intestine on CT, assisting with defining the colonic lumen and wall for more precise localization of PET uptake.

*Image Collection:* Immediately after contrast instillation, the animal was placed prone in the PET/CT scanner. Anesthesia was maintained with isoflurane (~1.5–2% in oxygen) via a nose cone throughout the scan session. The animal’s body temperature was maintained at ~37 °C via a heated pad, and respiration rate was continuously monitored. A low-dose CT scan was obtained for anatomical reference. CT parameters were 40–50 kVp and 200–500 µA with a 5-min acquisition time to cover the abdomen and pelvis (approximately 6–8 cm axial field of view). A static PET scan was performed at 1 h post-tracer injection. The PET acquisition used a single bed position covering the abdomen (and part of the thorax as needed) with a 30-min acquisition time for baseline and week 1 scans and up to 60 min for week 2 scans secondary to decreased disease activity. The PET data were reconstructed using ordered subset expectation maximization (OSEM) algorithms with scatter correction and attenuation correction. The typical voxel size was ~0.5 mm. The resulting PET images were automatically co-registered to the CT images using the direct scanner software. Image analysis: Analysis of PET/CT images was performed by utilizing PMOD software (ITK-SNAP 4.4.0) [33]. Using intraluminal contrast to outline the bowel, the colon was easily localized on the identified CT image. For each mouse, a volume of interest (VOI) was defined encompassing the entire colon (cecum to rectum). We semi-automatically segmented the bowel to the mid-sagittal line on CT using ITK-SNAP’s active contour tool and subsequently performed manual adjustments. This VOI was applied to the registered PET, and we recorded the mean standardized uptake value (SUV_mean_) within the colonic VOI. The SUV_mean_ was defined as the decay-corrected activity concentration (kBq/mL) in the VOI divided by the injected activity per body weight (kBq/g), producing a unitless value. Although we also recorded the maximum SUV (SUV_max_) in the colonic VOI for reference, we selected SUV_mean_ as our primary quantitative outcome measure to lower the effect of noise and focal hotspots. Finally, we confirmed, by checking the blood pool or background regions (heart or muscle), that there were no systemic differences in the distribution of the tracer between groups. To present the images, we generated fusion PET/CT images with rainbow color scales to denote the degree of increased PET uptake. All images were analyzed in a blinded manner with respect to treatment group to minimize bias. We confirmed the reproducibility of VOI placement by performing test–retest analysis on a subset of scans.

### 4.5. Histological Preparation

Upon completing the final imaging at the specified experimental endpoint (either one week or two weeks of treatment), mice were euthanized, and the entire colon was harvested for ex vivo analysis. The colon was harvested from the cecum to the rectum, gently flushed with PBS, and prepared as “Swiss rolls” for histology [34]. The process of preparing a Swiss roll included longitudinally slitting the colon and laying it flat with the mucosal side up. The colonic tissue was tightly rolled from the distal (the rectal end) to the proximal end around an applicator stick, thus creating a scroll with the mucosa facing inward. The rolled colon was fixed in 10% neutral buffered formalin (Azer Scientific, Morgantown, PA, USA) for 24–48 h at room temperature. Following fixation, samples were placed in 70% ethanol and stored at 4 °C until processing. Each Swiss-rolled colon was embedded in paraffin and sectioned transversally (so that the tissue appears as many concentric rings of colon on the slide). The Histopathology Core at Massachusetts General Hospital performed sample preparation of immunofluorescent staining for all samples, and they cut, fixed and stained 5 µm sections from each sample using standard protocols. For each mouse, sections from multiple levels of the colon roll were examined to ensure representative sampling of the proximal and distal colon.

### 4.6. Immunofluorescence Staining and Quantification

All prepared sections (5 µm thick) of paraffin-embedded colon tissue were subjected to immunofluorescence (IF) staining to measure Gzmb expression. The paraffin sections were first deparaffinized in xylene, followed by gradual rehydration from ethanol to water. The sections underwent antigen retrieval by boiling the slides for 20 min in Tris-EDTA buffer (10 mM Tris-base, 1 mM EDTA, and 0.05% Tween-20, at pH 9.0, Sigma Aldrich), before allowing them to cool to room temperature. Nonspecific binding sites were blocked by incubating the sections in blocking buffer (10% goat serum and 1% bovine serum albumin in TBS with 0.1% Triton X-100, ThermoFisher, Waltham, MA, USA) for 1 h. The sections were then incubated overnight at 4 °C in blocking buffer containing the primary antibody against granzyme B (Alexa Fluor^®^ 555 conjugated anti-granzyme B antibody [clone EPR22645-206], Abcam ab270743) at a 1:100 dilution. The following day, the slides were washed completely in TBS with 0.3% Triton X-100 and then applied with a Vector TrueView Autofluorescence Quenching Kit (Vector Laboratories) according to the manufacturer’s instructions, in order to diminish tissue autofluorescence (especially gut autofluorescence in the green channel due to lipofuscin or other pigments). The slides were then counterstained and mounted with DAPI-containing mounting medium (ThermoFisher), which labels nuclei with blue fluorescence. Fluorescence imaging was performed on an Agilent BioTek Cytation 5 Cell Imaging Multi-Mode Reader, using a 10× objective for overview images or 20× for more detail images. At least 5 random images were taken in DAPI and Alexa555 channels, representing each colon tissue section covering the mucosa and submucosa. For Gzmb IF quantification, the FIJI (ImageJ, version 1.54s) software was utilized. Images were converted to grayscale and total integrated fluorescence intensity was calculated for both the Gzmb channel (555 nm) and DAPI channel in each image. Then, to correct for cell number and section area, the ratio of Gzmb immunofluorescence signal to DAPI signal was calculated. The Gzmb/DAPI ratio represents the expression of granzyme B per cell (an estimate) for between-sample comparison. At least three non-overlapping images per tissue section were analyzed and averaged for each mouse, and the group means were determined. The quantification was performed in a blinded manner. In addition, the localization of Gzmb was qualitatively noted.

### 4.7. Bulk and Single-Cell RNA Sequencing and Analysis

To assess the molecular changes underlying imaging and histologic changes, we utilized bulk RNA sequencing (RNA-seq) performed on formalin-fixed paraffin-embedded (FFPE) colon tissues from each group (n = 3–4 mice per group per timepoint). FFPE scrolls (~50 µm thick) of colon FFPE tissues were prepared from the distal colon region of the Swiss rolls. A commercially available FFPE RNA isolation kit (e.g., Qiagen RNeasy FFPE kit) was used to extract RNA, and this isolation method included deparaffinization with xylene, proteinase K digestion, and DNA removal. The quantity and quality of RNA (RIN, DV200) were determined; despite FFPE samples giving shorter fragments, relative to acceptable standards (e.g., RIN > 7, DV200 > 60), all samples had enough RNA for the preparation of sequencing libraries after ribosomal RNA depletion. Sequencing libraries were constructed according to a low-input sequencing library protocol and used random priming to capture both coding and non-coding transcripts. Libraries underwent sequencing on an Illumina platform to produce 150 bp paired-end reads, yielding ~50 million reads per sample. Raw reads were trimmed for adapters and low-quality bases before alignment to the mouse reference genome (GRCm39) using the STAR aligner. Gene-level count matrices were generated (featureCounts) and normalized, and differential expression analysis was conducted in DESeq2 (Bioconductor in R) using our in-house RNAseq pipeline at https://github.com/arvinhm/RNAseqPipline, accessed on 29 April 2026. To be considered significant differential expression, we required an adjusted *p* < 0.05 (using a Benjamini–Hochberg false discovery rate (FDR) of 5%). To help deconvolute what cell types might be contributing to the differences seen, we leveraged published single-cell RNAseq data from DSS colitis models. We combined our bulk RNAseq data with an established single-cell reference dataset (GSE264408) profiling colon immune cells from healthy mice and DSS-induced acute versus chronic colitis [31]. Using the BayesPrism algorithm [35], we estimated the relative abundance of all cell types, including immune cells (e.g., T cells, NK cells, macrophages, etc.) for each bulk sample. This type of analysis indicated specific cellular changes associated with each treatment type. We also performed a pseudotime analysis using ScTour to order immune cells based on their activity status [36], as well as CellChat analysis of single cells to examine changes in cell–cell communication networks. In terms of the analyses presented in the paper, notably, interactions of cytotoxic lymphocytes (NK and cytotoxic T cells) through chemokine signaling (e.g., CCL5-CCR5 axes) were prevalent in colitis and predicted to be reduced with effective therapy [37].

### 4.8. Statistical Analyses

All quantitative data are presented as mean ± standard error of the mean (SEM) unless stated otherwise. For each of the group comparisons for imaging outcomes, (SUV_mean_), IF intensity, and histology scores were analyzed using appropriate statistical tests within GraphPad Prism 9. Where more than 2 groups were included in the analysis, we used one- or two-way ANOVA as appropriate followed by Tukey’s multiple comparisons test for individual pairwise comparisons. Repeated measures ANOVA was performed to investigate longitudinal imaging comparison over time within the same group (baseline vs. week 1 vs. week 2) and the Greenhouse–Geisser correction for repeated measures ANOVA was used if sphericity was violated. For comparisons between 2 groups (e.g., treated versus control at a timepoint), if data were approximately normally distributed, we used unpaired two-tailed Student’s *t*-tests; if normality was not appropriate, we used nonparametric Mann–Whitney tests instead. The threshold for determining significance was set as *p* < 0.05. As is needed for multiple comparisons, adjusted P values (FDR 5%) were reported.

## 5. Conclusions

In conclusion, this study establishes granzyme B-targeted PET imaging as a powerful tool for dynamic, non-invasive assessment of cytotoxic immune activity and therapeutic response in experimental colitis. We demonstrate that Gzmb PET can differentiate rapid responders (anti-TNFα) from suboptimal responders (prednisolone) and identify delayed response patterns (anti-IL-23) through distinct temporal imaging signatures. These patterns reflect underlying immunomodulatory mechanisms and are corroborated by tissue-level analyses. Our single-cell transcriptomic integration provides mechanistic insight into the cellular sources of Gzmb and the intercellular communication networks driving cytotoxic inflammation. Gzmb PET represents a paradigm shift toward precision imaging in IBD—moving beyond anatomical assessment to functional visualization of immune activity in real time. By revealing whether and when inflammation is truly controlled, Gzmb PET could enable more proactive, personalized disease management, sparing patients prolonged exposure to ineffective therapies while optimizing outcomes. While the rapid timescales and uniform disease progression of the murine model differ from chronic human disease, prior clinical data indicating Gzmb elevation in active human IBD suggest that the core principle of Gzmb as a dynamic biomarker holds translational promise. This work provides a preclinical proof-of-principle for Gzmb PET as a dynamic therapeutic monitoring tool and sets the stage for clinical validation studies. Prospective human trials evaluating Gzmb PET at defined intervals during therapy initiation will be necessary to determine whether the treatment-specific temporal signatures observed in this murine model translate to the more complex and protracted setting of human IBD.

## Data Availability

All data supporting the findings of this study are presented within the manuscript; raw data files are available from the corresponding author upon reasonable request.

## Abbreviations

IBD—Inflammatory Bowel Disease; UC—Ulcerative Colitis; CD—Crohn’s Disease; PET—Positron Emission Tomography; CT—Computed Tomography; Gzmb—Granzyme B; GZP—Granzyme B Peptide Inhibitor; DSS—Dextran Sulfate Sodium; IL—Interleukin; TNFα—Tumor Necrosis Factor alpha; NK—Natural Killer; IF—Immunofluorescence; Gzmb&E—Hematoxylin and Eosin; SUV—Standardized Uptake Value; VOI—Volume of Interest; ANOVA—Analysis of Variance; FDR—False Discovery Rate; IFNγ—Interferon gamma; Th—T helper; T_reg—Regulatory T Cell.
